# A tool to maximize air quality co-benefits while planning local climate actions: application to the Covenant of Mayors initiative

**DOI:** 10.12688/openreseurope.22919.1

**Published:** 2026-05-26

**Authors:** Fabio Monforti-Ferrario, Luana Valentini, Joana Bastos, Marta Giulia Baldi, Enrico Pisoni

**Affiliations:** 1 European Commission Joint Research Centre, Ispra, Italy; 2 Dukat Srl, Milan, Italy; 3 European Environment Agency, Copenhagen, Denmark

**Keywords:** Climate change, greenhouse gas mitigation, air quality, cities, Covenant of Mayors, co-benefits

## Abstract

This article presents a novel tool to quantify potential air quality co-benefits of local climate action and an analysis applying it to a large sample of cities across European and neighbouring countries. The tool estimates the effects of local actions tackling greenhouse gas (GHG) emissions associated with two sectors, namely residential buildings and transport, on air quality. It estimates emission changes associated with GHG mitigation actions described in city climate action plans (CAPs) for five key air pollutants, namely fine particulate matter (PM _2.5_), nitrogen oxides (NOx), sulphur dioxide (SO _2_), ammonia (NH _3_) and volatile organic compounds (VOC).

The analysis applies the tool to evaluate potential impacts of local climate action for 494 cities on ambient air pollutant emissions, based on 531 CAPs submitted to the Covenant of Mayors for Climate and Energy (CoM) initiative. The results demonstrate that GHG mitigation actions are often synergic with air pollution control strategies, offering significant air quality co-benefits. The magnitude and type of benefits depend on the sector and pollutant considered. Nevertheless, some actions may pose risks for air quality. There are risks of increasing, for example, ammonia emissions through a transport fuel switch and, more significantly, fine particulate matter emissions if solid biomass for heating in the residential sector is promoted. Therefore, context-specific data and case-by-case tailored analyses are needed to support the adequate integration of GHG mitigation and air quality policies at city level, avoiding potential trade-offs and maximizing synergies.

## Highlights

— Cities take an active role in climate change mitigation through key EU initiatives.
— A widely applicable tool is presented to evaluate air quality co-benefits.
— The analysis of 494 cities shows 2030 targets reduce GHG emissions by 30%.
— Greenhouse gas mitigation actions bring significant air quality co-benefits.
— In most cities, air pollutant reductions are larger than GHG reductions.
— Detailed analyses are needed when risks are identified, to avoid trade-offs.

## 1. Introduction

The European Union (EU) has set targets for progressively reducing its greenhouse gas (GHG) emissions with the aim of reaching climate neutrality by 2050. In 2008, acknowledging the role of local authorities in implementing successful climate policies, the Covenant of Mayors for Climate and Energy (CoM) initiative was launched. Initially, EU cities participating in the CoM had to submit a climate action plan (CAP) to reduce GHG emissions by at least 20% by 2020, in relation to 1990 levels. EU targets were updated in recent years and the CoM followed accordingly. In 2021, the CoM renewed its ambition in the EU, with participating cities pledging to the goal of climate neutrality by 2050, with an interim 55% emission reduction target by 2030 ( Melica et al., 2024).

The Joint Research Centre (JRC) of the European Commission provides methodological guidelines, technical and scientific support to CoM signatories. Since 2018, the JRC has brought to the attention of city administrations the importance of properly tuning climate change mitigation and air quality policies, to maximize synergies and avoid trade-offs. In this context, the JRC has also developed several tools and analyses on the evaluation of effects of local climate action on ambient air quality ( Monforti-Ferrario et al., 2018; Peduzzi et al., 2020). The tools aim to support cities in exploiting the synergies between GHG mitigation and air quality strategies and maximizing the co-benefits of their climate action plans.

Extensive literature has been published in recent years providing scientific evidence on the interplay between climate change mitigation and air pollution control strategies, mainly at global, country and regional levels. Climate change mitigation actions, reducing GHG emissions, can also have significant co-benefits for ambient air quality at the local scale ( Moutet et al., 2025). Studies have shown that many measures aimed at reducing carbon dioxide (CO _2_) emissions, for example, also reduce emissions of ambient air pollutants, such as particulate matter (PM), nitrogen oxides (NO _x_) and volatile organic compounds (VOCs), eventually resulting in improved air quality and health benefits ( Eric D. et al., 2022; Reis et al., 2022; Reynolds et al., 2024; WHO, 2025).

Nevertheless, limited evidence has been analysed at city level, and experience shows how cities need properly tailored tools to support the effective integration of climate and air quality policies ( Dyer et al., 2024). From a planning perspective, optimizing the air quality co-benefits of climate change mitigation actions could result in an important opportunity for local administrations engaged in reaching both their local climate targets and in complying with the increasingly stringent EU air quality legislation. Moreover, air quality and the related health co-benefits can increase acceptability and uptake of climate action ( Tonne et al., 2025). On the other hand, climate action can also pose risks to ambient air quality, and it is important to avoid unintended trade-offs in such cases ( Commane & Schiferl, 2022; Zauli-Sajani et al., 2024). Cities need to reinforce the dialogue and collaboration across departments with different responsibilities, and often with limited interaction, to ensure consistent climate change mitigation and air pollution policies ( Pilogallo et al., 2024; Ulpiani et al., 2024).

This article presents a novel tool to assess air quality benefits of local GHG mitigation actions in the transport and residential sectors. In a previous analysis, the JRC presented a tool for the retrospective evaluation of the air quality consequences of local climate actions based on the comparison of the so-called Baseline Emission Inventory (BEI) and a Monitoring Emission Inventory (MEI) for a group of CoM cities ( Peduzzi et al., 2020). With the new tool, we move towards a prospective evaluation of the air pollutant emissions rooted in the data included in city CAPs. In particular, this tool considers final energy use in the baseline year and the expected energy savings from GHG mitigation actions in the transport and residential sectors. In doing this, we have again fixed the BEI as the starting point, and we have estimated air pollutant emission changes associated with the actions foreseen. This tool is available online to cities using the ‘MyCovenant’ platform, and it is now made available for widespread (offline) use through a calculator excel template provided in supplementary materials.

The article is structured as follows: in section 2 we provide context on the linkages between local climate action and air quality in cities. Section 3 presents the methodology and main assumptions. In section 4 we present the application of the tool to CoM climate action plans (CAPs) to estimate co-benefits of planned GHG mitigation actions across EU cities. Lastly, brief concluding remarks are presented in section 5.

## 2. Background: Local climate action and air quality:

The European Green Deal has set the pathway to achieve climate neutrality by 2050, while at the same time pursuing ambitious pollution mitigation goals. In this context, the European Climate Law (Regulation (EU) 2021/1119) and the Zero Pollution Action Plan (COM/2021/400 final) established key targets for 2030, including a 55% reduction in GHG emissions and in premature deaths attributed to air pollution. Recently, the revision of the EU Ambient Air Quality Directive (Directive (EU) 2024/2881) also raised the air quality standards EU Member States should comply with from 2030.

Cities are at the forefront of climate change and air pollution mitigation across the EU. As they concentrate most EU population and economic activities, cities are associated with high energy requirements, GHG emissions and health impacts related to air pollution ( EEA, 2024; Li et al., 2026; Zhang et al., 2024). Since GHG and air pollutant emissions in cities often share common sources, in particular related to combustion of fuels in the transportation and buildings sectors, there is an evident interplay between local climate action and urban air quality.

In most cases, the linkages between GHG mitigation and air quality are synergistic: actions reducing final energy use or shifting from combustion of fossil fuels to electric systems, for example, which are central in local climate action, can offer significant air quality benefits. On a global scale, Shindell et al. (2018) showed how GHG emission reductions needed to move from a 2 °C to a 1.5 °C climate scenario would result in health co-benefits, quantified between 100 and 200 million human lives, until the end of the century, mostly due to reductions in O _3_ and PM _2.5_ emissions.

In China, a study on the transition to cleaner energy sources showed that promoting clean energy could reduce emissions of both SO _2_ and NO _x_ by more than 30% ( Bo et al., 2023). Liu et al. (2024) developed a synergy index between CO _2_ and PM _2.5_ reductions for Chinese cities. An analysis of a 3-year action plan targeting GHG and air pollutant emissions in a sample of Chinese cities estimated a reduction of CO _2_ of only 1% between 2017 and 2020, but up to 17 and 25% for air pollutant emissions, namely PM _2.5_ and SO _2_ ( Shu et al., 2022). However, the analysis showed that the industrial sector offered larger co-benefits, while transport and energy sectors offered limited co-benefits. Recently, Li et al. (2026) focused on the health co-benefits arising from carbon emission mitigation in the transport sector of a Chinese city. The research applied emission factors for a baseline and projected years to estimate emission changes for CO _2_, PM _2.5_ and O _3_, in alternative mitigation scenarios.

In Europe, Tonne et al. (2025) presented a framework to evaluate health co-benefits of climate change mitigation, including those related to air pollution, and highlighted the need for further research and tools, in particular to complement analysis using Integrated Assessment Models (IAMs) at national level evaluating implemented mitigation actions, with evidence to support local action.

Air quality risks have also been identified, for example, when local climate action promotes the use of biomass for energy ( Commane & Schiferl, 2022; Zauli-Sajani et al., 2024). In order to avoid trade-offs and maximize air quality co-benefits, local climate and air quality strategies should be integrated, and cities need tools to inform integrated action. In this context, the JRC has brought to the attention of local administrations the importance of properly tuning climate change mitigation and air quality policies ( Ulpiani et al., 2024). Moreover, it has developed several tools to support cities involved in two major urban climate change mitigation initiatives, namely the Global Covenant of Mayors for Climate and Energy and the EU Mission for 100 Climate-neutral and smart cities. Cities participating in the initiatives can access the tool through dedicated online interfaces. While air quality co-benefits are not explicitly mandated among the objectives of the CoM or Cities Mission, the integration of GHG emission mitigation and air quality strategies has been promoted.

The JRC has also performed several analyses on potential air quality co-benefits and trade-offs of local climate actions undertaken by cities in the CoM. Monforti-Ferrario et al. (2028), investigated air pollution emissions abated by energy saving actions planned in climate action plans. Peduzzi et al. (2020) performed an evaluation of the air quality consequences of local climate action based on the comparison between energy use and GHG emission data from climate action plans and from monitoring reports (i.e., a retrospective evaluation, after action implementation). These analyses showed potential air quality co-benefits and trade-offs of implemented actions, and they highlighted the importance of tools that can support cities while developing their climate strategies and GHG mitigation actions, i.e., informing on potential synergies and expected co-benefits during the development and planning of their local climate actions.

Mission cities have also shown to be generally aware of the interplay between local climate action and air quality, however, in most climate action plans air quality remains treated at a generic and qualitative level, and quantitative assessments or evaluations are seldomly performed. Most action plans in the Cities Mission (which have been analysed by the JRC) pose no evident risks or concerns in relation to ambient air quality, but cities expect and sometimes count on air quality co-benefits. Researchers and practitioners have also highlighted that air quality co-benefits play an important role in stakeholder engagement and in increasing acceptance and uptake of local climate action, and monetised benefits of climate action often outweigh economic costs of climate policies ( Moutet et al., 2025). Moreover, since many cities currently face challenges to comply with the revised Ambient Air Qualitive Directive, there is an immediate opportunity to strengthen the integration and maximize synergies between local climate action and air quality strategies.

This article presents the latest air quality tool developed by the JRC in the context of the CoM, which estimates changes in air pollutant emissions associated with GHG mitigation actions. The tool provides an ex-ante evaluation, i.e., during the development and planning of actions, before implementation. In particular, it presents the tool’s underlying methodology, a stand-alone calculator excel template in supplementary materials for wider (offline) application, and an analysis applying the tool to 494 EU cities participating in the CoM. It contributes to advance research and practice by providing a consistent, simple and widely applicable tool to model GHG and air pollutant emissions in integrated manner and by providing a consistent and harmonized analysis of air quality co-benefits of local climate action for a large sample of EU cities, based on reported data.

## 3. Materials and methods

Under the CoM and Cities Mission frameworks, as in other reference initiatives in Europe and worldwide, cities develop climate action plans (CAP) that include (i) a GHG emission inventory for a baseline reference year and (ii) a set of actions to reduce annual GHG emissions by a target year ( Davide et al., 2025). The baseline emission inventory (BEI) provides a quantitative evaluation of the ‘starting point’ in the CAP, against which progress achieved through GHG mitigation actions is monitored and benchmarked. Typically, a city GHG inventory is developed by combining local data on energy use in different sectors (by energy source/carrier) with GHG emission factors calculated for the corresponding energy source/carrier. Regarding the actions, cities usually estimate the energy savings and GHG emission reduction that they expect to achieve by actions, either single or aggregated by sector (i.e., the overall GHG emission reduction expected from a set of actions tackling GHG emissions in a selected sector, such as transport).

As such, CAPs include (i) final energy use and GHG emissions by sector by energy source/carrier in the baseline year and (ii) energy saving and GHG reduction estimates of the actions by sector. Essentially, the final energy use data is a matrix of energy use by sector and by energy source/carrier, which cities compile as activity data used then to calculate GHG emissions by applying corresponding emission factors.

In brief, the new air quality tool draws on the final energy use data by sector and energy source/carrier in the baseline year, which is multiplied by GHG emission factors (EFs) to develop the BEI, and it combines the same data with air pollutant EFs to estimate air pollution emissions in the baseline year. Then, it applies pollutant emission factors to the actions’ expected energy savings in the target year to estimate air pollutant emission changes resulting from the local climate actions foreseen. The methodology draws on the same approach and methodological principles applied in the previous tool ( Peduzzi et al., 2020), and the new tool was developed for two sectors, namely residential buildings and transport. These are dominant sectors accounting for a major share of GHG and air pollutant emissions in cities and, therefore, they are included in the CoM key sectors for which all cities shall report activity and GHG emission data ( Bastos et al., 2025; Davide et al., 2025).

**Figure 1.**
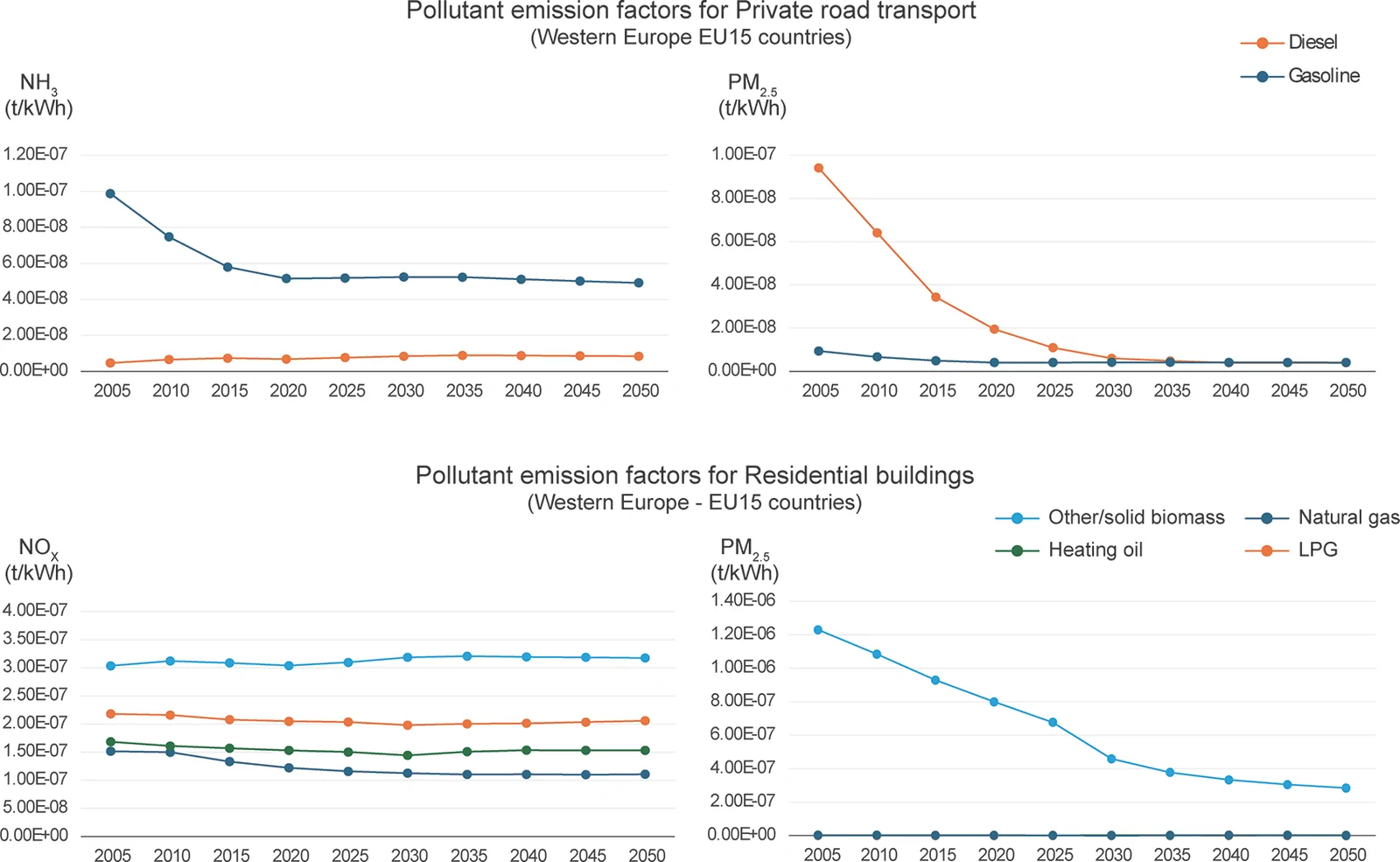
Example of average air pollutant emission factors in t/kWh for Private road transport and Residential buildings by energy source/carrier in Western Europe for 2005–2050. Own elaboration based on GAINS data.

Specifically, the tool multiplies the final energy use for each sector-carrier by emission factors (EFs) for five air pollutants, namely PM _2.5_, NO _x_, SO _2_, NH _3_ and VOC. The EFs for the five pollutants for the sector-carrier combinations have been obtained from GAINS projections ( Amann et al., 2011). GAINS provides data on air pollutant emissions and energy/fuel consumption for all countries of continental Europe and for other neighbour countries, including Iceland, Turkey, Azerbaijan, Armenia and Georgia, and cover a time span from 2005 to 2050, in 5-year steps. We calculated EFs for the two selected sectors, five air pollutants and four regions, namely Western Europe, Central and Eastern Europe, Western Balkans and Others, as the ratio between overall emissions and energy use. Considering regions (i.e., groups of countries) flattens EFs avoiding larger uncertainties and it helps overcoming limited data availability for smaller countries. Examples of average air pollutant EFs for Western Europe are shown in Figure 1. It is worth noticing that, although smoothed by the regional approach used, a certain grade of irregularity remains in EF temporal evolution, with some cases of small fluctuation, as it is for instance the case of biomass related NOx emissions in the building sector. In the calculation of air pollutant EFs, a mapping step was performed to establish correspondence between GAINS and inventory sectors and fuels (used in the CoM), which is available in supplementary materials, together with the country groups considered.

To estimate changes in air pollutant emissions in the (future) target year, we considered data on local climate actions reported in the CAP, namely the expected energy savings. Since data provided is often limited to overall energy savings and GHG emission reduction for the sector in the target year, without insight on reduction by energy source/carrier, assumptions are needed on the future energy mix. The tool applies two alternatives to the energy mix evolution, referred to as ‘pathways’. In one pathway, labelled as Baseline Energy mix, the tool assumes the same energy mix shares over time, i.e., the shares for each energy source/carrier from the baseline year are applied to the target year. In other words, the energy savings are applied proportionally to each energy source/carrier in the baseline energy mix. In the other pathway, labelled as MinGHG Energy mix, we consider that energy sources/carriers with higher GHG emission factors are targeted first. As such, the energy savings are sequentially applied to reduce consumption of the fuel with highest GHG EF first, and if energy savings are larger than the use of that fuel, its consumption is reduced to zero and the remaining savings are applied to the second most GHG intensive fuel, and so on. This pathway considers a maximized GHG reduction per energy savings. The GHG EFs by energy carrier/fuel used in this sequential selection are those provided by cities in their CAP. The CoM data collection provides GHG EFs for local energy and grid-supplied national electricity use that can be used by cities in their GHG inventories ( Bastos et al., 2024).

The use of two pathways is a simplification needed to overcome data limitations. Local climate actions implemented by cities can affect final energy use and the evolution of the energy mix in a sector in different ways, depending on their action portfolio. As such, the air quality tool results should be seen as a reference range of a likely interval of possible outcomes. The supplementary materials include an illustrative example of the application of the air quality tool, including the two pathways.

The excel calculator provides an alternative to the online tool, allowing wider application by cities that have no access to the online ‘MyCovenant’ platform. To use this calculator, inputs on final energy use in the baseline year, GHG emission factors and estimated energy savings expected from the GHG mitigation actions for the residential buildings and transport sectors should be introduced by users, and the tool automatically calculates air pollutant emission changes in the target year.

### 3.1 Tool application: preliminary validation of the methodology

In order to test our methodology and to perform a preliminary validation of the tool, we applied it to a large sample of cities from Europe and neighbouring countries participating in the CoM. Data from climate action plans (CAPs) submitted to MyCovenant is publicly available in a comprehensive dataset, published and maintained by the JRC ( Franco et al., 2024). Our analysis used the 4 ^th^ release of the CoM dataset ( Baldi et al., 2023). It is worth noting that the JRC performs a set of consistency checks before accepting CAPs, and it regularly assesses the robustness of the CoM dataset as a whole, investigating and, if necessary, eliminating outliers before publishing each dataset release.

From the dataset we selected all cities with a minimum population of 50 000 inhabitants and an accepted/validated CAP, and excluded 4 records in which the reported GHG emission reduction for a sector (residential buildings or transport) exceeded its the baseline emissions. This resulted in the final selection of 494 cities and 531 CAPs. As mentioned, cities provide final energy use and GHG emissions in the baseline for each sector-carrier combination and expected energy savings and GHG emission reductions by sector (i.e., for all actions planned for that sector). This data was used to calculate air pollutant emission changes and to relate these changes to the expected GHG emission reductions, for the residential building and transport sectors, as provided by cities.

The CAPs were divided into two groups. The first group (denoted as T20) includes CAPs with a GHG mitigation target for the year 2020. Since the target year in for this group is now a past year, the analysis does not anticipate potential co-benefits, but it serves as a reference for comparison with the results of the second group of CAPs, with more ambitious GHG mitigation targets. T20 contains 338 cities in 33 countries for a total population of more than 63 million. More than 90% of these cities selected a baseline year between 2005 and 2015, with the remaining ones choosing an older baseline (up to 1990).

The second dataset (denoted as T30+) includes CAPs with mitigation targets for the year 2030 (and in some cases also targets beyond 2030). It contains 195 CAPs in 25 countries for a total population of more than 49 million. A relatively large number of CoM signatories are in the process of moving from previous 2020 targets to updated targets for 2030 and beyond ( Melica et al., 2022). Even in this case, more than 90% of signatories have chosen a baseline year equal or more recent than 2005, up to 2021 although the remaining ones still prefer an older baseline, even in this case back up to 1990. It is also worth noticing that 39 signatories have presented both a SECAP targeting 2020 and another successive one targeting 2030 or beyond, so they appear in both the datasets.

## 4. Results and discussion

**Figure 2.**
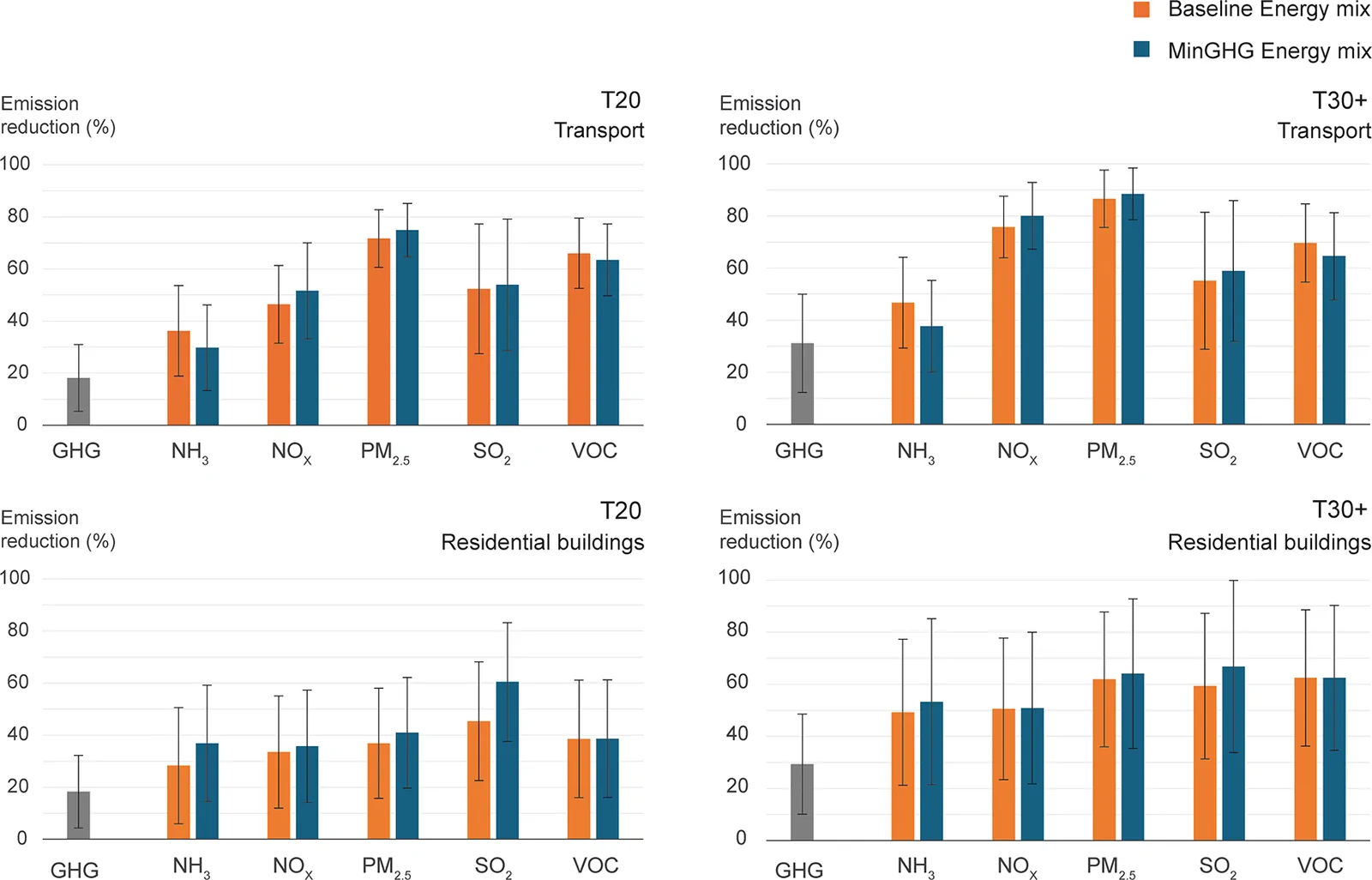
Average emission decrease (%) for GHG (in grey) and the studied pollutants in the BEI Energy mix (orange) and MinGHG Energy mix (blue) pathways in the transport (upper row) and residential (lower row) sectors for climate action plans (CAPs) targeting 2020 (T20, on the left) and 2030 or beyond (T30+, on the right). Error bars represent the standard deviation.

Figure 2 shows the estimated average percentage emission decrease for GHG emissions (in grey) and for the five air pollutants studied in the two key sectors of transport and residential heating and in the two pathways developed, marked as Baseline Energy mix and MinGHG Energy mix respectively, for the CAPs in the T20 and T30+ datasets.

**Figure 3.**
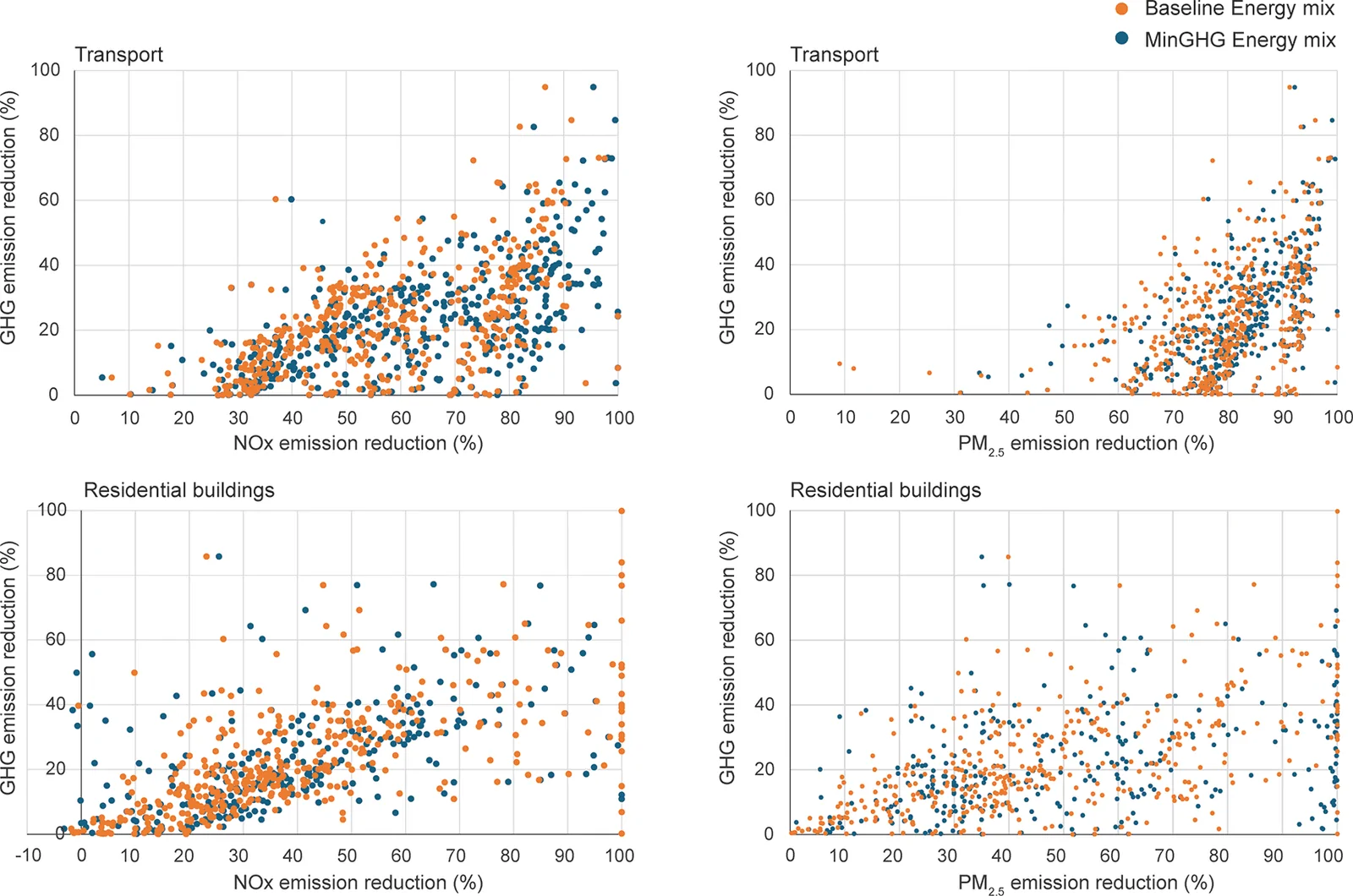
Scatter plots for GHG and air pollutant emissions reductions (in % for NO _x_ and PM _2.5_) in the transport (upper row) and residential buildings (lower row) sectors for the Baseline Energy mix (orange) and MinGHG Energy mix (blue) pathways. Each point represents a climate action plan (CAP), either targeting 2020 (T20) or 2030 and beyond (T30+).

As expected, T30+ shows high average emission reductions of both GHG and air pollutants than T20, reflecting local climate action with higher GHG mitigation targets is also contributing to lower air pollutant emissions. Figure 2 also includes the standard deviation of the percentage emission changes, represented in the form of error bars. The quite important size of the standard deviation indicates a large variability of reduction across cities. Such variability is even more evident in Figure 3, which shows a set of scatter plots reporting GHG and air pollutant emission reductions for NO _x_ and PM _2.5_ in the two sectors, again distinguishing between Baseline and MinGHG Energy mix pathways. Since the chart relates air pollutant and GHG reductions and it intends to highlight the overall variability and diversity of cities, the scatter plots include CAPs from both T20 and T30+ datasets.

The results, as illustrated in Figures 2 and 3, show:

- CO_2_ emissions. CoM cities have properly pursued GHG emission reductions in the CAPs with 2020 targets and are also planning relevant GHG mitigation actions for the future ( Figure 2). The average GHG emission reduction value of about 30% in both transportation and residential sectors CAPs with 2030+ targets are fully in line with the analysis in Melica et al. (2022).
- Air pollutant emission reductions. On average, for CAPs with 2030+ targets, air pollutant emission reductions (in percentage) are significantly more substantial than GHG emission reductions. While the GHG emission reduction is similar in both sectors (29 and 31% for buildings and transport, respectively), ambient air pollutant emission reductions are larger in the transport sector. In the residential sector, average air pollutant emission reductions range from 43 to 64%, for NH _3_ and VOC, respectively; while in the transport sector they range from 48 to 89%, for NH _3_ and PM _2.5_, respectively.
- Air pollutant emissions decrease for a twofold reason. Firstly, as a result of the decreased energy use planned by cities. On top of this, most air pollutant emission factors show decreases over time, sometimes very important, linked to improved technology and stricter legislation (see Figure 1). Comparing T20 and T30+ in Figure 2, we see that the more ambitious targets set for GHG mitigation for the year 2030 and beyond also bring lager reductions in air pollutant emissions, showing an overall positive synergy between climate change mitigation and air quality.
- Cross-city variability. The variability across the sample cities of both GHG and air pollution emission reductions is significant. This indicates a wide variety of approaches and measures across the cities investigated which are free to put emphasis on any on the two sectors analysed, or both, or again to choose to focus their efforts on other sectors not included in this study.
- Comparing energy mix pathways. In most cases, air pollutant emission reductions achieved in the MinGHG Energy mix pathway are larger than in the Baseline Energy mix pathway. However, in some cases the difference is very small. This is consistent with the observation that, in general, fuels with lower GHG emission factors also have lower air pollutant emissions (e.g., for coal and natural gas). Figure 2 shows nevertheless a noticeable exception in the case of NH _3_ in the transport sector, where the GHG EFs for gasoline fuelled cars is about 10% lower than diesel ( Bastos et al., 2024) while the opposite is true for NH _3_ EFs, with gasoline cars having higher NH _3_ emissions than diesel (see Figure 1). It is worth highlighting, however, that the contribution of the transport sector to NH _3_ emissions is generally low, compared to other sectors/sources (e.g., agriculture).
- Sectorial comparison. Figure 2 shows how air pollutant emission reductions are on average more relevant in the transport sector than in the residential one. This is again consistent with the development of effective EURO technologies and legislation, especially when one considers that in some cases the baseline emission inventories refer to several years in the past, and since then major progresses have been achieved in reducing air pollutant emissions.
- Trade-offs. Although in general air pollutant emissions decrease similarly or even more than GHG emissions, Figure 3 shows some cases where this does not happen, including a few cases in the residential sector where air pollution emissions (slightly) increase. This effect could be caused by either an increase of EFs in time (see again Figure 1 and related text) or, by an increased relative share of fuels causing higher air pollutant emissions in the energy mix. A typical case is biomass-based fuels in the residential sector: indeed, biomass is often accounted having no CO _2_ emissions (carbon neutral perspective), while it can have significant air pollutant emissions (which are accounted for with high EFs). Again, the presence of CoM cities relying on biomass fuels to achieve their climate targets is consistent with previous analyses and findings ( Peduzzi et al., 2020). These cities should pay specific attention to the undesired consequences of their GHG mitigation strategy as far as air pollution is concerned. For instance, fuel switch towards biomass for residential heating should be limited to districted heating and high efficiency’ boilers and stoves with air pollution control to mitigate ambient air pollution trade-offs.

The methodology and data used in our tool have intrinsic limitations that may affect the robustness of results. First, the future emission factors applied in the target year are associated with high uncertainty, which results from the use of projected data on fuel use and emissions. In the future, previous projections for now past years could be compared to EFs calculated retrospectively, to provide insight on the potential significance of this uncertainty and how it may affect the results. Moreover, while the use of average EFs for groups of countries reduces fluctuations resulting from uncertain and limited data for small countries, different groupings would result in different EFs applied in the tool. Second, other assumptions on the emissions associated with energy use may affect the results, such as those adopted in the two pathways to overcome limited information on the target year energy mix. While context-specific data could significantly improve the robustness of results, requiring further details for the tool might limit a wide application at an early action planning stage. Nonetheless, researchers and practitioners are strongly advised to perform context-specific case-by-case analyses to fully exploit their direct knowledge of their urban context.

Lastly, our tool estimates air pollutant emission changes, but health-related co-benefits depend on the transport, dispersion and transformation of pollutants in the urban atmosphere, and the resulting concentrations and population exposure. The assessment of health co-benefits associated with the emission changes estimated by the tool would be a valuable advance for practitioners.

## 5. Conclusions and outlook

This article presents a novel tool to evaluate air quality co-benefits during local climate action planning and an analysis of a large sample of European cities. In summary, our analysis confirms and underlines previous results using monitored data that CoM cities will continue to achieve air quality co-benefits while reducing GHG emissions, with some sectors and pollutants offering larger synergies than others. Nevertheless, some cases of trade-offs still emerge, demonstrating the need for tools and context-specific data to support the development of integrated strategies tackling climate change and air pollution in cities. For example, the use of solid biomass fuels should be carefully addressed to mitigate impacts on air quality and public health.

It is worth highlighting that this is one of the first widely applicable tools to quantitatively estimate air quality co-benefits of local climate action, relying on reference and validated analytical tools such as GAINS. The tool is generally easy to use and provides immediate results without needing additional data to the ones normally provided in a typical CAP. Nonetheless, reliable and robust evaluation of air quality co-benefits and risks arising from local climate action depends on quality context-specific data. The tool has been developed under the CoM framework, and it assumes the user has relevant knowledge and data for the territory. For users with limited data and knowledge, the tool might request high data collection requirements.

Our experience in discussing synergies and trade-offs between local climate and air pollution action with local administrations across the EU shows that there is still a need to raise awareness across their political and technical governing bodies interactions between the often-different departments dealing with air pollution and climate mitigation often need to be facilitated and we think the new tool can support and enable a better integration in local policymaking.

Following the dissemination of the tools, an important next step is the analysis of health co-benefits. In fact, while the methodology presented here estimates changes in air pollutant emissions, going from air pollutant emission changes, to concentrations, population exposure and the result public health impacts is paramount to maximize co-benefits and increase the uptake of local climate actions. In the future, a comparative analysis of the expected GHG reductions and air quality co-benefits associated with local climate action with monitoring data could provide further insight on the robustness of results. Lastly, it be worth further investigating the main drivers of variability across cities, to provide context-specific policy recommendations.

## Use of generative AI

During the preparation of this work the authors used GPT@JRC (model GPT OSS 120B) to revise and refine small parts of drafted text. After using the tool, the authors reviewed and edited the content as needed and take full responsibility for the content of the publication.

## Ethics and consent

Ethical approval and consent were not required for this study.

## Data Availability

This study used the Covenant of Mayors dataset (4 ^th^ release):
— Baldi, Marta; Franco de Los Rios, Camilo; Melica, Giulia; Treville, Aldo; Bertoldi, Paolo (2026): GCoM - MyCovenant, 4th Release - March 2023. European Commission, Joint Research Centre [Dataset] doi:10.2905/JRC.05BR6RG PID: http://data.europa.eu/89h/b425918f-53a1-495c-8619-cd370c302eb0 Supplementary materials, including additional methodological details and results, the air quality tool excel-calculator, a version of the calculator with filled data (as an example) and the SECAP analysis data are available on zenodo:
— Monforti-Ferrario, F., Valentini, L., Bastos, J., Baldi, M.G. and Pisoni, E. (2026) A tool to maximize air quality co-benefits while planning local climate actions: application to the Covenant of Mayors initiative - Supplementary materials. doi:10.5281/zenodo.18635572 The dataset and supplementary materials are available under the terms of the Creative Commons Attribution 4.0 International (CC BY 4.0).
