# Supplementary material for: A tool to maximize air quality co-benefits while planning local climate actions: application to the Covenant of Mayors initiative
Source: Open Res Eur. 2026 May 26;6:180. [Version 1] doi: 10.12688/openreseurope.22919.1 (PMC13562957; doi:10.12688/openreseurope.22919.1)
Supplement: Peer review history

# Reviewer report: Reviewer response for version 1

**DOI:** 10.21956/openreseurope.24783.r78250

**Published:** 2026-09-29

**Authors:** Antonio Piersanti

**Affiliations:** 1 ENEA – Italian Agency for New Technologies, Energy and Sustainable Economic Development, Bologna, Italy

The article evaluates the quantitative reduction of air pollutants emissions as a co-beneficial consequence of reduction of emission of greenhouse gases, at city level, based on the dataset of the Covenant of Mayors for Climate and Energy initiative of the European Union.

 The article is clearly written and organized. The topic of synergies and trade-offs of policy action tackling emissions of climate forcers and atmospheric pollutions is of paramount interest. Therefore, though methods and tools in the article do not introduce novel science, the quantitative insight on the Convenient of Mayors figures is of relevant interest for informed practical application of emission reduction measures.

 A small number of typos and wrong references is present, which I suggest to correct after a careful revision of the text.

Is the study design appropriate and does the work have academic merit?

Yes

Is the work clearly and accurately presented and does it cite the current literature?

Yes

If applicable, is the statistical analysis and its interpretation appropriate?

Yes

Are all the source data underlying the results available to ensure full reproducibility?

Yes

Are the conclusions drawn adequately supported by the results?

Yes

Are sufficient details of methods and analysis provided to allow replication by others?

Yes

Reviewer Expertise:

integrated assesment modeling of emissions and air quality, air quality observation

I confirm that I have read this submission and believe that I have an appropriate level of expertise to confirm that it is of an acceptable scientific standard.

# Reviewer report: Reviewer response for version 1

**DOI:** 10.21956/openreseurope.24783.r79680

**Published:** 2026-09-10

**Authors:** M. Omar Nawaz

**Affiliations:** 1 Cardiff University, Cardiff, UK

**Major Comments**

 This manuscript by Monforti-Ferrario et al. presents a novel tool for exploring how climate action plans in European cities could affect local air pollution emissions for two major polluting sectors: transport and residential. Overall, this paper is clearly written, well presented, and has interesting and relevant visualisations that characterise the model results across the cities that participate in the CoM initiative. I have no major comments for this manuscript, and I would recommend this manuscript for indexing once a few minor comments are addressed that I have indicated below.

**Minor Comments**

- Introduction, Paragraph 3: When discussing climate mitigation and air quality co-benefits it could be useful to highlight the direct connection of CH4 and tropospheric O3 to further evidence this point.
- Background, Paragraph 1: Would it be possible to include the specific standard values (e.g., XX ug/m3) in addition to saying that the EU has raised them? Then you might be able to comment on what percentage of cities in the CoM are above these standards still
- Background, Paragraph 3: I would suggest revising “O3 and PM2.5 emissions” to “O3 and PM2.5 precursor emissions” as the prior implies direct emission of O3
- Methods Paragraph 3: What is included in “transport”? This is often a sector that can differ from study to study (e.g., some include shipping/aircraft emissions). From Table 1 of the supplement, it appears that this is just vehicles though?
- Methods Paragraph 3: I do not agree with the statement that “These are dominant sectors accounting for a major share of GHG and air pollutant emissions in cities” unilaterally because there can be significant city-to-city variability in the dominant sources. For example, in industry hubs, industrial/energy generation sources could supplant transport and residential emissions as the dominant sectors. Or the same could be said for major shipping hubs. Perhaps you could soften the language in the statement here?
- Methods Paragraph 4: Do you know which components are included in primary PM _2.5_? For example, BC, OC, Fugitive Dust, etc.
- Methods Paragraph 4: It might be worth stating somewhere here or a prior paragraph that you are using annual emissions given that some sources, such as residential, have seasonal-regional trends in their magnitude.
- Figure 1: Can I ask why you have selected NH _3_ instead of NO _x_ for Figure 1 for private road transport? I think NO _x_ would be more relevant to the associated health burden when considering the transport sector specifically (although NH _3_ is relevant as well).
- Figure 3: Perhaps you could also indicate the 2020 vs 2030 cities by shape? For example circle could represent 2020 and square could represent 2030?
- Conclusion, Paragraph 4: I agree that extending this tool to include public health impacts would be quite useful; however, there will be some more challenges in doing so (e.g., long-range transport, non-linear secondary chemistry, seasonality of air pollutant formation, projected population changes). I am curious how you might address this, but of course, that is not specifically relevant to this manuscript.

Is the study design appropriate and does the work have academic merit?

Yes

Is the work clearly and accurately presented and does it cite the current literature?

Yes

If applicable, is the statistical analysis and its interpretation appropriate?

Yes

Are all the source data underlying the results available to ensure full reproducibility?

Yes

Are the conclusions drawn adequately supported by the results?

Yes

Are sufficient details of methods and analysis provided to allow replication by others?

Yes

Reviewer Expertise:

My area of expertise relevant to this publication is urban air quality modelling and source-receptor assessment.

I confirm that I have read this submission and believe that I have an appropriate level of expertise to confirm that it is of an acceptable scientific standard.

# Reviewer report: Reviewer response for version 1

**DOI:** 10.21956/openreseurope.24783.r75822

**Published:** 2026-06-27

**Authors:** Sabrina Bresciani

**Affiliations:** 1 Politecnico di Milano Dipartimento di Design, Milan, Lombardy, Italy

The paper is informative and provides relevant pragmatic insights. It presents a tool for estimating the air-quality co-benefits and possible trade-offs of local climate mitigation actions. The article contributes both a methodological tool and an application to a large sample of Covenant of Mayors climate action plans.

 The focus on residential buildings and transport is relevant. The application to 494 cities provides a substantial empirical basis for the analysis. The article also usefully distinguishes between co-benefits and possible trade-offs.

 While the pragmatic contribution is emphasized and the policy context is clearly provided, the manuscript would be strengthened by sharpening its analytical and theoretical contribution, for instance by linking more explicitly to the scholarly literature.

 The analysis applies the tool to a large dataset, but is not fully clear why the term validation is used since the paper does not appear to compare predicted emission changes against observed outcomes. More information should also be provided on the rational for the classification of countries into the specific four selected European regions.

 It would be beneficial to clarify for readers what the novel contribution of the new tool is compared to previous JRC tool mentioned in the manuscript.

 The following sentence would benefit from rephrasing to improve clarity and to better qualify the basis of the claim: “Our experience in discussing synergies and trade-offs between local climate and air pollution action with local administrations across the EU shows that there is still a need to raise awareness across their political and technical governing bodies interactions between the often-different departments dealing with air pollution and climate mitigation often need to be facilitated and we think the new tool can support and enable a better integration in local policymaking.” 

 The reference to Monforti-Ferrario et al. (2028) appears to be a typo: in the reference list this is reported as Monforti-Ferrario et al. (2018). The year and capitalization should be corrected.

 Overall, the paper presents a relevant tool, applies it to a substantial sample of city climate action plans, and offers useful insights into both co-benefits and possible trade-offs.

Is the study design appropriate and does the work have academic merit?

Yes

Is the work clearly and accurately presented and does it cite the current literature?

Partly

If applicable, is the statistical analysis and its interpretation appropriate?

Yes

Are all the source data underlying the results available to ensure full reproducibility?

Yes

Are the conclusions drawn adequately supported by the results?

Yes

Are sufficient details of methods and analysis provided to allow replication by others?

Yes

Reviewer Expertise:

Experiments, mixed methods, social innovation, systemic design, sustainability, Mission-oriented innovation

I confirm that I have read this submission and believe that I have an appropriate level of expertise to confirm that it is of an acceptable scientific standard.
